# Exome sequencing in routine diagnostics: a generic test for 254 patients with primary immunodeficiencies

**DOI:** 10.1186/s13073-019-0649-3

**Published:** 2019-06-17

**Authors:** Peer Arts, Annet Simons, Mofareh S. AlZahrani, Elanur Yilmaz, Eman AlIdrissi, Koen J. van Aerde, Njood Alenezi, Hamza A. AlGhamdi, Hadeel A. AlJubab, Abdulrahman A. Al-Hussaini, Fahad AlManjomi, Alaa B. Alsaad, Badr Alsaleem, Abdulrahman A. Andijani, Ali Asery, Walid Ballourah, Chantal P. Bleeker-Rovers, Marcel van Deuren, Michiel van der Flier, Erica H. Gerkes, Christian Gilissen, Murad K. Habazi, Jayne Y. Hehir-Kwa, Stefanie S. Henriet, Esther P. Hoppenreijs, Sarah Hortillosa, Chantal H. Kerkhofs, Riikka Keski-Filppula, Stefan H. Lelieveld, Khurram Lone, Marius A. MacKenzie, Arjen R. Mensenkamp, Jukka Moilanen, Marcel Nelen, Jaap ten Oever, Judith Potjewijd, Pieter van Paassen, Janneke H. M. Schuurs-Hoeijmakers, Anna Simon, Tomasz Stokowy, Maartje van de Vorst, Maaike Vreeburg, Anja Wagner, Gijs T. J. van Well, Dimitra Zafeiropoulou, Evelien Zonneveld-Huijssoon, Joris A. Veltman, Wendy A. G. van Zelst-Stams, Eissa A. Faqeih, Frank L. van de Veerdonk, Mihai G. Netea, Alexander Hoischen

**Affiliations:** 1 Department of Human Genetics, Radboud University Medical Center, Nijmegen, The Netherlands; 2 Department of Genetics and Molecular Pathology, Centre for Cancer Biology, SA Pathology and the University of South Australia, Adelaide, South Australia Australia; 3 Department of Pediatrics, Children’s specialist Hospital, King Fahad Medical City, Riyadh, Saudi Arabia; 4 Department of Medical Biology, Faculty of Medicine, Akdeniz University, Antalya, Turkey; 5 Department of Pediatric immunology, Pediatrics, Radboud University Medical Center, Nijmegen, The Netherlands; 6 Department of Pediatric Hematology and Oncology, Comprehensive Cancer center, King Fahad Medical City, Riyadh, Saudi Arabia; 7 Radboud Expertise Center for Immunodeficiency and Autoinflammation, Department of Internal Medicine, Radboud University Medical Center, Nijmegen, The Netherlands; 8 Department of Pediatric Infectious Diseases and Immunology, Wilhelmina Children’s Hospital, University Medical Center Utrecht, Utrecht, The Netherlands; 9 Department of Genetics, University of Groningen, University Medical Center Groningen, Groningen, the Netherlands; 10 Princess Máxima Center for Pediatric Oncology, Utrecht, the Netherlands; 11 Department of Pediatric Rheumatology, Pediatrics, Radboud University Medical Center, Nijmegen, The Netherlands; 12 Department of Clinical Genetics, Maastricht University Medical Center+, Maastricht, The Netherlands; 13 PEDEGO Research Unit and Medical Research Center Oulu, University of Oulu, Oulu, Finland; 14 Department of Clinical Genetics, Oulu University Hospital, Oulu, Finland; 15 Department of Hematology, Radboud University Medical Center, Nijmegen, The Netherlands; 16 Department of Clinical Immunology, Maastricht University Medical Center, Maastricht, The Netherlands; 17 Department of Clinical Science, Department of Informatics, Computational Biology Unit, University of Bergen, 5020 Bergen, Norway; 18 Department of Clinical Genetics, Erasmus MC, University Medical Center, Rotterdam, The Netherlands; 19 Department of Pediatrics, School for Nutrition and Translational Research in Metabolism (NUTRIM), Maastricht University Medical Center+, Maastricht University, Maastricht, The Netherlands; 20 Institute of Genetic Medicine, Newcastle University, Newcastle-upon-Tyne, UK; 21 Department of Human Genetics and Department of Internal Medicine, Radboud University Medical Center, P.O. Box 9101, 6500 HB Nijmegen, The Netherlands

**Keywords:** Routine diagnostics, Genetic diagnosis, Exome sequencing, Primary immunodeficiencies

## Abstract

**Background:**

Diagnosis of primary immunodeficiencies (PIDs) is complex and cumbersome yet important for the clinical management of the disease. Exome sequencing may provide a genetic diagnosis in a significant number of patients in a single genetic test.

**Methods:**

In May 2013, we implemented exome sequencing in routine diagnostics for patients suffering from PIDs. This study reports the clinical utility and diagnostic yield for a heterogeneous group of 254 consecutively referred PID patients from 249 families. For the majority of patients, the clinical diagnosis was based on clinical criteria including rare and/or unusual severe bacterial, viral, or fungal infections, sometimes accompanied by autoimmune manifestations. Functional immune defects were interpreted in the context of aberrant immune cell populations, aberrant antibody levels, or combinations of these factors.

**Results:**

For 62 patients (24%), exome sequencing identified pathogenic variants in well-established PID genes. An exome-wide analysis diagnosed 10 additional patients (4%), providing diagnoses for 72 patients (28%) from 68 families altogether. The genetic diagnosis directly indicated novel treatment options for 25 patients that received a diagnosis (34%).

**Conclusion:**

Exome sequencing as a first-tier test for PIDs granted a diagnosis for 28% of patients. Importantly, molecularly defined diagnoses indicated altered therapeutic options in 34% of cases. In addition, exome sequencing harbors advantages over gene panels as a truly generic test for all genetic diseases, including in silico extension of existing gene lists and re-analysis of existing data.

**Electronic supplementary material:**

The online version of this article (10.1186/s13073-019-0649-3) contains supplementary material, which is available to authorized users.

## Background

Primary immunodeficiencies (PIDs) are genetically and phenotypically heterogeneous disorders characterized by an inborn increased susceptibility to infections. From the genetic perspective, over 300 genes have been identified as monogenic causes of PIDs [1–4]. The majority of pathogenic variants in PID genes are reported to cause disease in a purely autosomal recessive (AR) fashion (69%), compared to an autosomal dominant (AD) (20%), AR and AD (5%), and X-linked (XL) (6%) manner [1–4].

The phenotype of PID patients ranges from frequent or more severe relatively common infections to serious clinical manifestations due to rare pathogens that require immediate clinical care to prevent fatality [2]. In addition to infections, some patients with PIDs can also experience autoimmune or inflammatory conditions, as well as malignancy and developmental abnormalities [1, 2]. PIDs are divided in 10 specific subtypes according to the International Union of Immunological Societies (IUIS) PID classification [1]. The clinical variable phenotype of PIDs makes diagnosing patients based on their respective phenotypes challenging. A recent publication describes that 55% of 110 cases were misdiagnosed based on their initial clinical characteristics [5]. In order to prevent this, a more robust and rapid identification of the underlying genetic defect would be of great clinical benefit: a “genotype-first approach” may provide a molecularly defined diagnosis in a significant amount of cases.

Genetic diagnosis of PIDs has been available for a relatively long time, but until now it has been complicated by the need to pinpoint the gene of interest: invariably, this is linked to the correct (and often difficult) clinical diagnosis in the first place. Fortunately, the availability of rapid and cheap sequencing methodologies now allows for more unbiased genetic diagnostics. Exome sequencing in particular has been shown to be an effective tool to elucidate the genetic defect underlying other types of heterogeneous disorders [6, 7]. We performed exome sequencing to provide a genetic diagnosis for patients suffering from a broad range of immune deficits. The identification of the genetic basis of PIDs provides insight into the molecular mechanisms of these diseases and may offer customized treatment options [5, 8–10]. Compared to targeted enrichment approach, exome sequencing has several major advantages: first, the in silico exome gene panel can easily be adjusted upon identification of novel PID genes; second, exome-wide analysis allows analysis for variants in novel genes not included in the gene panel; third, exome sequencing allows genome-wide data access and hence more reliable detection of copy number variants (CNVs) and regions of homozygosity (ROH) [5, 11–14]. To reduce the complexity of the analysis and speed up the process, exome sequencing can be combined with an in silico analysis of a set of already known disease genes [15].

Due to large genetic and phenotypic heterogeneity of PIDs, and the rapidly increasing number of PID genes identified over the last years [1, 2, 16], we implemented exome sequencing as a single test in routine diagnostics for PIDs in 2013 in a large tertiary academic hospital (Radboud University Medical Center). From then until October 2016, a group of 254 consecutively referred patients suffering from PIDs have been tested by exome sequencing, and here we report on their genetic diagnostic outcome.

## Methods

### Samples

Between May 2013 and October 2016, 254 patient DNA samples (249 families) from the main referring clinics for exome sequencing to our diagnostic laboratory (160 from The Netherlands, 8 from Finland, and 81 from Saudi Arabia) were submitted for whole exome sequencing. The average age at testing was 21 years (range from 1 month to 79 years), and the male/female distribution was 117M/137F (details in Additional file 1: Table S1). Families were counseled and provided consent for “PID gene panel only” or “gene panel and exome-wide analysis” as presented here.

### Exome sequencing procedure

Genomic DNA was isolated from whole blood. The experimental workflow of all exomes was performed at BGI Europe (Beijing Genome Institute Europe, Copenhagen, Denmark). Exonic regions were enriched using the Agilent (Agilent Technologies, CA, USA) SureSelect V4 (*n* = 85) or V5 (*n* = 169) kit and sequenced using an Illumina Hiseq (Illumina, CA, USA) sequencer with 101-bp paired end reads to a median coverage of > 75x. Sequenced reads were mapped to the hg19 reference genome using the mapping algorithm from BWA [17] (version 0.5.9-r16) and called by the GATK unified genotyper [18] (version 3.2-2). All variants were annotated using an in-house pipeline for exome analysis containing variant and gene-specific information, amongst which the variant population frequencies from > 5000 in-house exomes [7].

### Exome variant interpretation

For the gene panel analysis, a bioinformatic in silico filter was applied to select for variants affecting the known > 300 PID genes [19]. This gene panel consisted of 263 established (OMIM) PID genes in 2013, expanding to 302 genes in 2016 (all earlier versions available) [19]. Variants were filtered for coding, non-synonymous variants with population frequencies below 1% in our in-house database (a database of > 5000 exomes), and evaluated regarding their possible pathogenicity. The latter was performed using population frequencies [20], nucleotide conservation scores (PhyloP), and in silico pathogenicity predictions (SIFT, Polyphen2, Mutationtaster) combined with genetic and phenotypic overlap with earlier described cases to estimate the contribution of the genetic variant to disease [21].

Eighty-one percent of diagnosis-negative patients provided consent for exome-wide analysis. All variants derived from exome sequencing were prioritized for coding, non-synonymous variants with population frequencies of ≤ 1% in-house and ≤ 5 homozygous occurrences reported in EXAC for autosomal recessive candidates, and allele counts of ≤ 10× in house or ≤ 20× in EXAC for autosomal dominant candidates [20]. The exome-wide analysis focused on variants in recently described genes and genes involved in immune pathways, based on GO terms, mouse knockout model phenotypes, or the Kyoto Encyclopedia of Genes and Genomes (KEGG). In addition, we filtered for variants in genes with known NCBI protein-protein interactions with known disease genes for similar phenotypes [22].

All identified genetic variants were judged on their possible pathogenicity based on guidelines of the Association for Clinical Genetic Science and the American College of Medical Genetics and Genomics [21, 23]. We only considered variants disease-causing if we found sufficient phenotypic overlap with earlier described cases based on OMIM [24]. In addition, we only report variants classified as class 5 (pathogenic), class 4 (likely pathogenic) (see Table 1 and Additional file 2: Table S2), or class 3 (uncertain significance) (see Additional file 3: Table S3), because the variants classified as class 2 (likely benign) or class 1 (benign) are probably tolerated [21, 23].

**Table 1:** Expected disease, molecular diagnosis, and potential treatment options for PID patients with diagnoses

Solved European cases									
Patient ID	Referred from	Gender	Age	Clinical diagnosis/expected disease	Mutation inheritance	ACMG variant class	Mutation(s) identified	Treatment options	Ref treatment
134.1	Finland	Female	4	ADA2 deficiency	AR (hom)	5/5	CECR1 p.(R169Q/R169Q)	Anti-TNF treatment	[40]
1.1	Netherlands	Female	50	APECED	AR (hom)	5/5	AIRE p.(R257*/R257*)		
217.1	Netherlands	Female	23	Chronic granulomatous disease	AR (hom)	5/5	NCF2 p.(Y293*/Y293*)	* Specific prophylaxis bacterial and fungal (IFN-γ treatment) consider HSCT	[43, 44]
70.1	Netherlands	Female	27	Ciliary diskinesia	AR (hom)	4/4	RSPH9 p.(M1T/M1T)	Possibility for lung transplantation due to diagnosis of PCD.	[34]
46.1	Finland	Female	15	Chronic mucocutaneous candidiasis	AD	4	STAT1 p.(Q243E/wt)	Ruxolitinib; consider HSCT; IgG replacement therapy	[30–32]
149.1	Netherlands	Female	55	Chronic mucocutaneous candidiasis	AD	5	STAT1 p.(Q271P/wt)	Ruxolitinib; consider HSCT; IgG replacement therapy	[30–32]
222.1	Netherlands	Male	29	Complement deficiency	AR (hom)	5/5	C7 p.(G379R/G379R)	* Prophylaxis: vaccination against meningococcus	[46]
103.1	Netherlands	Male	48	CVID	AD	5	NFKB1 p.(S302fs/wt)	IgG replacement therapy	
116.1	Netherlands	Male	52	CVID	AR (hom)	5/5	CECR1 p.(L503fs/L503fs)	Anti-TNF treatment	[40]
169.1	Netherlands	Male	57	CVID, malignancies	XL	4	MAGT1 p.(S24*)	IgG replacement therapy Mg supplement therapy: Clinical trial NCT02496676	[49]
227.1	Netherlands	Male	57	Familial cold autoinflammatory syndrome	AD	5	NLRC4 p.(S445P/wt)	Anti-IL-1 treatment	[53]
32.1	Netherlands	Female	11 months	Hermansky-Pudlak syndrome	AR (CH)	4/4	AP3B1 p.(K59fs/D613fs)		
76.1	Netherlands	Female	29	HSV infections	AD	5	GATA2 p.(R86fs /wt)	HSCT	[35]
142.1	Netherlands	Female	20	Hyper IgE syndrome	AD	5	CFTR p.(W1282*/wt)		
162.1	Netherlands	Male	9	IgG deficiency	AD	5	TNFRSF13B p.(C104R/wt)		
213.1	Netherlands	Male	3 months	Interstitial lung disease	AR (hom)	4/4	DHFR p.(G21R/G21R)	Folinic acid treatment	[51]
213.2	Netherlands	Female	1	Unknown (affected sibling 213.1)	AR (hom)	4/4	DHFR p.(G21R/G21R)	Folinic acid treatment	[51]
33.1	Netherlands	Male	53	Joint, skin, upper respiratory tract infections	AD	4	CXCR4 p.(S343fs/wt)	Plerixafor; CXCR4 antagonist future treatment option	[29]
69.1	Netherlands	Male	9	Kabuki syndrome	AD	5	KMT2D p.(E5425K/wt)		
29.1	Netherlands	Female	28	PAPA syndrome	AD	5	PSTPIP1 p.(E250K/wt)	Anti-IL-1 treatment	[28]
220.1	Netherlands	Female	16	Recurrent infections, IFN-γ deficiency	AD	4	CARD11 p.(T43P/wt)	Glutamine supplementation (IFN-γ treatment)	[50]
					AD	4	MEFV p.(M680I/wt)	Colchicine anti-IL-1 treatment	[52]
173.1	Netherlands	Female	12	Recurrent urticaria	AD	5	NLRP1 p.(L332fs/wt)	Anti-IL-1 treatment	
52.1	Netherlands	Male	4	Shwachman-Diamond	AD	5	TERC n.(37A>G/wt)		
159.1	Finland	Male	1	X-linked thrombocytopenia	XL	5	WAS p.(V75M)	HSCT	[39]
Solved Saudi Arabian cases									
202.1	Saudi Arabia	Female	7	Autoimmune lymphoproliferative syndrome	AD	5	CARD11 p.(G123S/wt)	Glutamine supplementation; IFN-γ treatment	[50]
147.1	Saudi Arabia	Female	6	Autoimmune lymphoproliferative syndrome, anti-HCV	AR (hom)	4/4	CASP8 p.(A155S/A155S)		
					AD	5	CBL c.(1228-2A>G/wt)		
83.1	Saudi Arabia	Male	2	Bare lymphocyte syndrome II	AR (hom)	5/5	RFXANK p.(D121V/D121V)	HSCT	[37]
					AD	5	INSR p(R145C/wt)		
106.1	Saudi Arabia	Male	5 months	Bare lymphocyte syndrome II	AR (hom)	5/5	RAG1 p.(K186fs/K186fs)	HSCT	[8]
185.1	Saudi Arabia	Female	8 months	Bare lymphocyte syndrome II	AR (hom)	5/5	RFX5 p.(V378fs/V378fs)	HSCT	[37]
148.1	Saudi Arabia	Male	6	Complement deficiency	AR (hom)	5/5	C8A p.(Y210*/Y210*)	* Prophylaxis: vaccination against meningococcal disease	[46]
129.1	Saudi Arabia	Female	8 months	Chronic granulomatous disease	AR (hom)	5/5	CYBA c.(58+4-7del/58+4-7del)	* Specific prophylaxis bacterial and fungal (IFN-γ treatment); consider HSCT	[43, 44]
161.1	Saudi Arabia	Male	2	Chronic granulomatous disease	AR (hom)	5/4	CYBA p.(A117E/A117E)	* Specific prophylaxis bacterial and fungal (IFN-γ treatment); consider HSCT	[43, 44]
165.1	Saudi Arabia	Female	8	Chronic granulomatous disease	AR (hom)	5/5	CEBPE p.(R135*/R135*)	Consider anti-inflammatory therapy	
168.1	Saudi Arabia	Male	3	Chronic granulomatous disease	XL	5	CYBB p.(E347fs)	* Specific prophylaxis bacterial and fungal (IFN-γ treatment); consider HSCT	[43, 44]
156.1	Saudi Arabia	Female	3	Congenital neutropenia, myelofibrosis	AR (hom)	4/4	VPS45 p.(L410P/L410P)	HSCT	[47]
113.1	Saudi Arabia	Female	13	Dyskeratosis congenita	AR (hom)	4/4	WRAP53 p.(R387C/R387C)		
122.1	Saudi Arabia	Female	19	Gray platelet syndrome	AR (hom)	5/5	ITGA2B p.(R1026W/R1026W)		
126.1	Saudi Arabia	Female	11	Hypogammaglobulinemia	AR (hom)	5/5	DNMT3B p.(V836M/V836M)	Consider HSCT; IgG replacement therapy	[41]
127.1	Saudi Arabia	Female	10	Hypogammaglobulinemia, bronchiectasis	AR (hom)	5/5	ZBTB24 p.(Q498fs/Q498fs)	Consider HSCT; IgG replacement therapy	[42]
127.2	Saudi Arabia	Female	12	Hypogammaglobulinemia	AR (hom)	5/5	ZBTB24 p.(Q498fs/Q498fs)	Consider HSCT; IgG replacement therapy	[42]
138.1	Saudi Arabia	Male	1	Hypogammaglobulinemia	AR (hom)	5/5	AK2 p.(A182D/A182D)	Consider HSCT; IgG replacement therapy	[45]
138.2	Saudi Arabia	Female	4	Hypogammaglobulinemia	AR (hom)	5/5	AK2 p.(A182D/A182D)	Consider HSCT; IgG replacement therapy	[45]
189.1	Saudi Arabia	Female	4	Hypogammaglobulinemia	AR (hom)	5/5	DNMT3B p.(V836M/V836M)	Consider HSCT; IgG replacement therapy	[41]
189.2	Saudi Arabia	Male	1	Hypogammaglobulinemia	AR (hom)	5/5	DNMT3B p.(V836M/V836M)	Consider HSCT; IgG replacement therapy	[41]
196.1	Saudi Arabia	Male	7	Hypogammaglobulinemia	AR (hom)	5/5	DNMT3B p.(V836M/V836M)	Consider HSCT; IgG replacement therapy	[41]
198.1	Saudi Arabia	Male	2	Hypogammaglobulinemia	AR (hom)	5/5	JAK3 p.(R403H/R403H)	HSCT	[8]
204.1	Saudi Arabia	Male	6 months	Hypogammaglobulinemia	AR (hom)	5/5	DNMT3B p.(V836M/V836M)	Consider HSCT; IgG replacement therapy	[41]
100.1	Saudi Arabia	Female	8	IgG deficiency	AD	5	PIK3R1 c.(1425+1G>T/ wt)	IgG replacement therapy	[38]
186.1	Saudi Arabia	Male	6	Microcytic anemia	AD	4	HBB p.(Q7V/wt)		
236.1	Saudi Arabia	Male	6	Non-immune hemolytic anemia	XL	5	G6PD p.(V461G)	* Dietary: Avoidance of fava beans and specific drugs	[54]
240.1	Saudi Arabia	Female	2 months	Pancytopenia	AR (hom)	5/5	MTHFD1 p.(R173C/R173C)	Folic acid and folinic acid treatment	[51]
94.1	Saudi Arabia	Female	8	Pancytopenia, hyper- and hypogammaglobulinemia	AR (CH)	5/3	FANCA p.(L910fs/C1142Y)		
114.1	Saudi Arabia	Female	8 months	SCID	AR (hom)	4/4	DCLRE1C p.(P117Q/P117Q)	Consider HSCT	[8]
115.1	Saudi Arabia	Female	8 months	SCID	AR (hom)	4/4	ZAP70 p.(S524C/S524C)	HSCT	[8]
105.1	Saudi Arabia	Male	5 months	SCID, HLH	XL	5	IL2RG p.(I273fs)	Consider HSCT, IgG replacement therapy	[8]
112.1	Saudi Arabia	Male	8 months	SCID, Omenn syndrome	AR (hom)	5/5	RAG1 p.(K186fs/K186fs)	HSCT	[8]
146.1	Saudi Arabia	Male	11 months	SCID, HLH	AR (hom)	5/5	JAK3 Ex10 Deletion	HSCT	[8]
154.1	Saudi Arabia	Male	3 months	SCID, BCGitis	AR (hom)	4/4	RAG2 p.(K106E/K106E)	HSCT	[8]
199.1	Saudi Arabia	Male	3	SCID, Burkitt’s lymphoma	AR (hom)	4/4	LCK p.(R480fs/R480fs)	HSCT	[8]
84.1	Saudi Arabia	Male	9	Severe eczema	AD	4	SAMHD1 p.(F329fs/wt)	Consider anti-IL-5 or anti-IL4R treatment	
61.1	Saudi Arabia	Female	12	Severe infections, pancytopenia	AD	4	CTLA4 p.(G146R /wt)	Abatacept (recombinant CTLA4)	[33]
82.1	Saudi Arabia	Female	4	Severe infections, thrombocytopenia	AR (hom)	5/5	LRBA p.(T1587fs/T1587fs)	Abatacept (recombinant CTLA4)	[36]
190.1	Saudi Arabia	Male	4	Severe lung infections	AR (hom)	5/5	AK2 p.(A182D/A182D)	Consider HSCT; IgG replacement therapy	[45]
145.1	Saudi Arabia	Female	5 months	Severe infections, hypergammaglobulinemia	AR (hom)	5/5	CFTR c.(579+1G>A/579+1G>A)		
239.1	Saudi Arabia	Female	4 months	Severe infections, hemolytic anemia	AD	4	ANK1 p.(Q1313*/wt)		
242.1	Saudi Arabia	Female	4	Severe infections, leukocytosis, hypergammaglobulinemia	AD	5	STAT3 p.(V713M/wt)	* Specific prophylaxis bacterial and fungal (IFN-γ treatment)	[32]
160.1	Saudi Arabia	Female	2	Shwachman-Diamond, CD3 deficiency	AR (hom)	4/4	PRF1 p.(R410P/R410P)	Possible T cell gene therapy (under development)	[48]
153.1	Saudi Arabia	Male	15	T cell acute lymphoblastic leukemia	AR (hom)	5/5	NBN p.(Y197fs/Y197fs)		
					AD	4	RPL5 p.(G140S/wt)		
107.1	Saudi Arabia	Male	21	Thrombocytopenia	XL	5	WAS p.(T48A)	HSCT	[39]
188.1	Saudi Arabia	Male	6 months	TORCH	AR (hom)	4/4‡	RNASEH2B p.(D119G/D119G)		
195.1	Saudi Arabia	Male	1 months	Transaldolase deficiency	AR (hom)	5/5	TALDO1 p.(Q265fs/Q265fs)		
193.1	Saudi Arabia	Male	38	Viral infections, autoimmune manifestations, thrombocytopenia	AR (hom)	5/5	C7 p.(G378R/G378R)	* Prophylaxis: vaccination against meningococcus	[46]

Table 1 lists clinical diagnoses and identified pathogenic or likely pathogenic genetic mutations in all 72 patients from Europe and Saudi Arabia. In addition, the table provides potential therapeutic options resulting from identification of the molecular defect

*AD* autosomal dominant, *APECED* autoimmune polyendocrinopathy-candidiasis-ectodermal dystrophy, *AR* autosomal recessive, *CH* compound heterozygous, *CVID* common variable immune deficiency, *HSCT* hematopoietic stem cell transplantation, *HLH* hemophagocytic lymphohistiocytosis, *hom* homozygous, *IFN-γ* interferon- γ, *IL-1* interleukin-1, *IgG* immunoglobulin G, *PCD* primary ciliary dyskinesia, *SCID* severe combined immunodeficiency, *TNF* tumor necrosis factor, *TORCH* toxoplasmosis, other, rubella, cytomegalovirus, and herpes simplex infections, *XL* X-linked

*Indirect measures or prophylaxis

### Homozygosity calling

Regions of homozygosity (ROH) were called using RareVariantVis [13]. Downstream filtering included filtering for larger (≥ 5 Mb) homozygous regions, in which ≥ 85% of all variants were called to be homozygous.

### CNV calling

Copy number variant (CNV) calling was performed using CoNIFER to calculate RPKM-based absolute *Z*-scores [14, 25]. Rare copy number variants affecting PID-associated genes were followed up similarly as the earlier described single nucleotide variants (SNVs), small insertions, or deletions (indels).

### Validation of detected variants and follow-up in families

All reported low-quality variant calls (GATK quality by depth < 500) were confirmed by standard Sanger sequencing. Patients with reported class 3 or class 4 variants were counseled to perform further analyses on their respective families. To gain more genetic evidence for causality of the variants, we have performed co-segregation analysis to confirm de novo mutations or carrier status in parents.

### Immunophenotyping

For 75% of patients, the immunophenotypes were further characterized by determining one or more of the functional immunological defects. This included quantification of cellular subtypes and antibodies in whole blood, and measurement of cytokine production capacity upon in vitro stimulation assays. The latter experiments were performed similar to previous reports [26]. In brief, peripheral blood mononuclear cells (PBMCs) were isolated by density centrifugation and cultured with a medium or a medium supplemented with immune response-inducing ligands or heat-killed pathogens. Cytokine production capacity was measured using an enzyme-linked immunosorbent assay (ELISA).

## Results

### Patient cohort

**Fig. 1:**
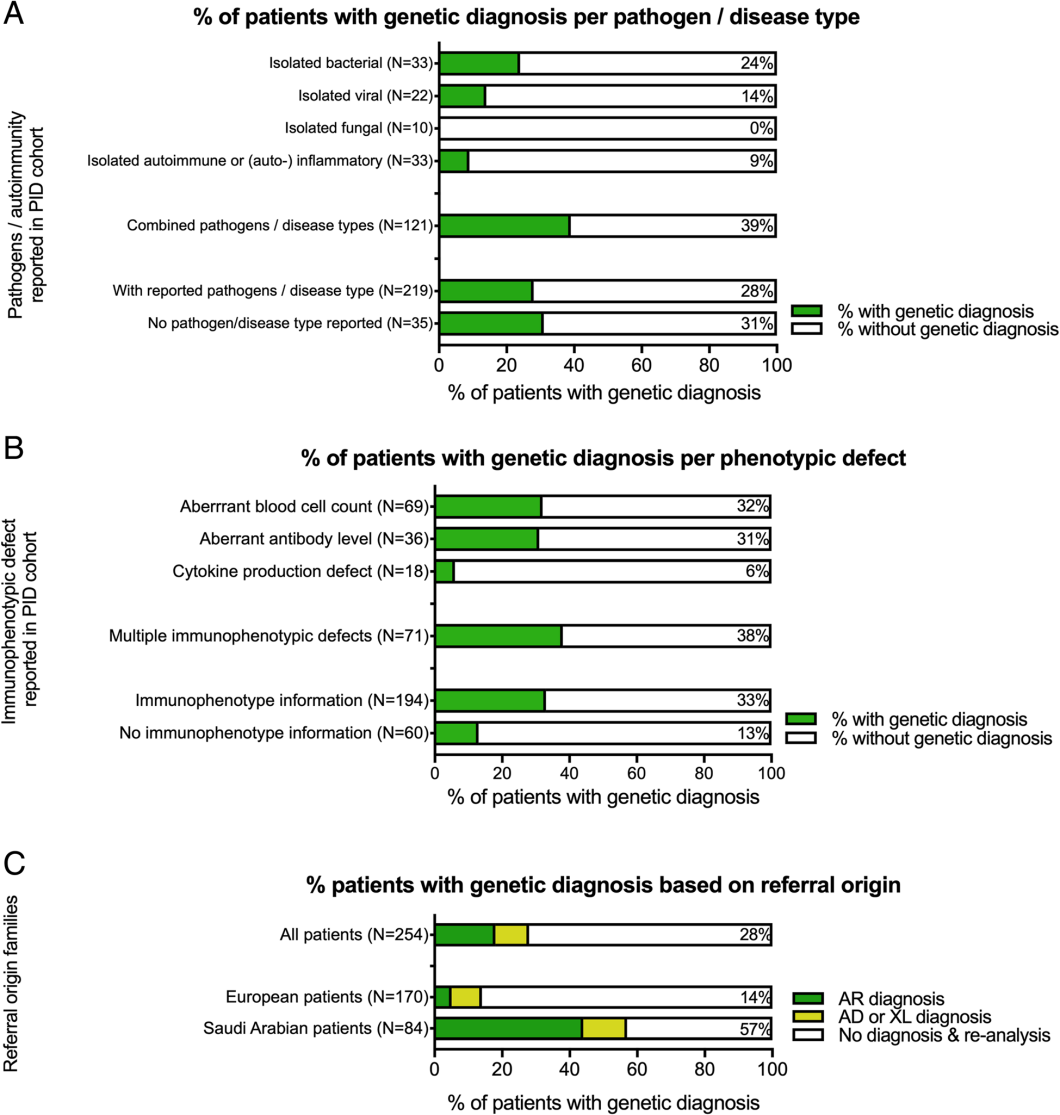
Clinical and immunophenotypic overview of the 254 patients included in the diagnostic PID cohort, including percentages of patients with genetic diagnoses per subgroup. **a** For 219 patients, pathogens and/or autoimmunity was identified. **b** Immunophenotypic defects were characterized in 194 patients. Quantification of blood cell numbers, antibody levels, and cytokine production aided to determine the genetic diagnosis for these patients. **c** The diagnostic yield per cohort based on the country from which the patients were referred. Compared to European patients, a higher percentage of patients from Saudi Arabia received a genetic diagnosis

In total, 254 patients from 249 families were referred for diagnostic exome sequencing. Two hundred nineteen patients presented with unusual bacterial, viral, or fungal infections or autoimmune manifestations or combinations of such (Fig. 1a, Additional file 1: Table S1 and Additional file 4: Table S4). Immunophenotype defects were observed in 194 patients; of those, 133 patients had aberrant blood cell counts, 102 patients showed altered antibody profiles, and 31 patients revealed irregular cytokine production (Fig. 1b, Additional file 1: Table S1 and Additional file 4: Table S4).

### Exome sequencing

Whole exome sequencing resulted in an average coverage of 120.7× (Agilent SureSelect V4) and 130.2× (Agilent SureSelect V5), covering 95.3% of the exome at least 20-fold. For the genes within our gene panel, the average coverage was 132.6× and 93% of the base pairs of these genes were covered at least 20 times (details in Additional file 5: Table S5).

### Exome variant interpretation

**Fig. 2:**
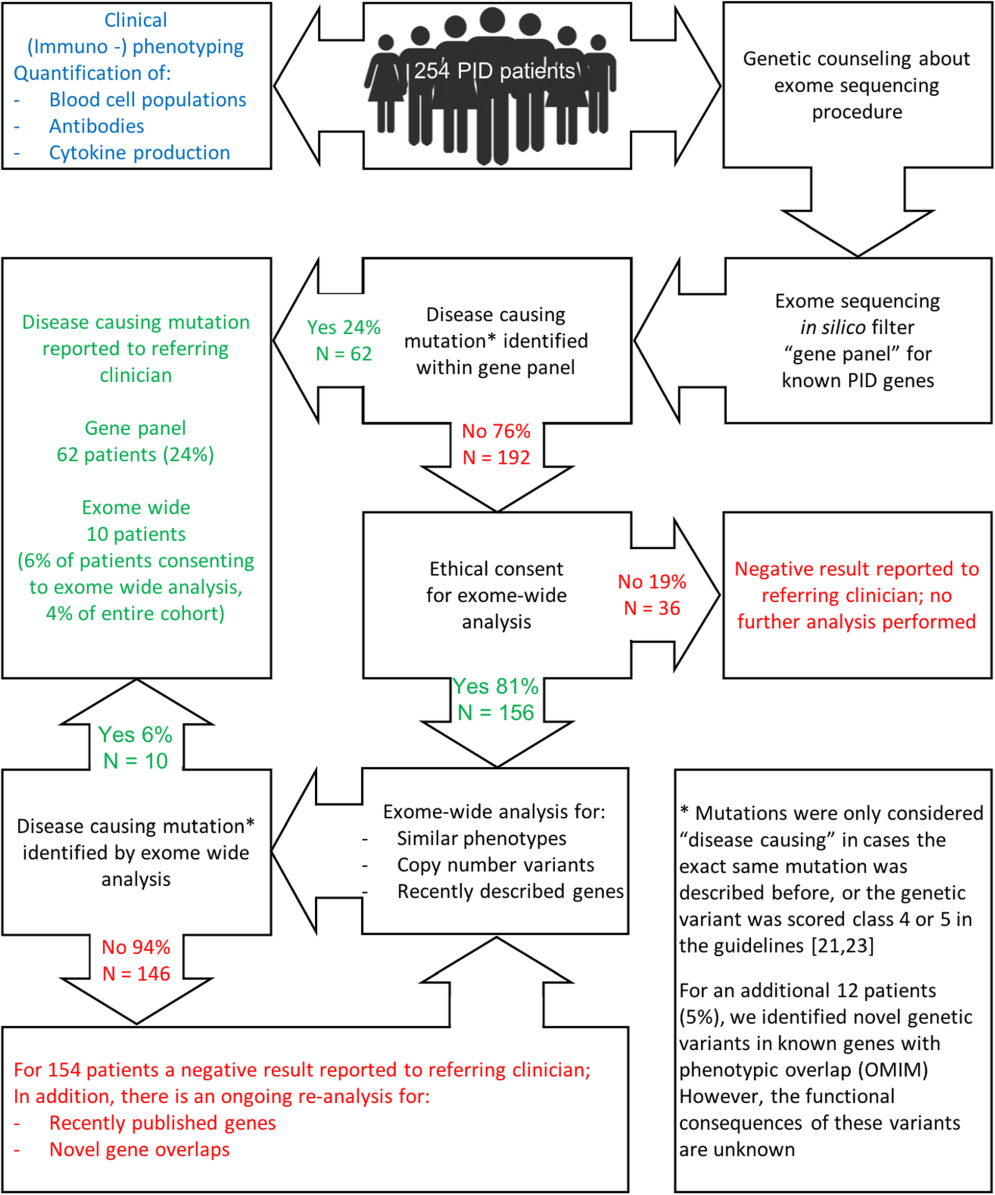
Schematic flowchart overview of the diagnostic exome procedure. Two hundred fifty-four patients from 249 families were referred for exome sequencing. Gene panel analysis resulted in a genetic diagnosis for 24% of patients. Eighty-one percent of diagnosis-negative patients provided consent for exome-wide analysis of their data. This analysis resulted in a genetic diagnosis for 10 additional patients (6% of exome-wide analyzed patients, 4% of the entire cohort). Data of the remaining 146 patients are re-analyzed for analysis of novel and recently published genes

For each exome, a bioinformatic in silico panel of genes was applied as a first-tier test to select for variants affecting the > 300 known PID genes [19]. This list is regularly updated in silico whenever novel PID genes are discovered. This yielded on average 1542 genetic variants in known PID genes per individual. Additional filtering for coding, non-synonymous variants and population frequency ≤ 1% resulted in 10 to 40 variants per case, which were evaluated on their possible pathogenicity (Fig. 2). Pathogenic (class 4 or class 5) variants were identified in at least one of the known PID genes for 62 patients (24%). Eighty-one percent of genetic-diagnosis-negative patients provided consent for exome-wide analysis. (Re-)analysis for variants in recently published PID genes and genes causing defects in immunological sub-pathways yielded an additional (class 4 or class 5) genetic diagnosis for 10 patients (5% of all exome-wide analyzed samples; 4% of the entire cohort). The combined result of our two-step analysis provided a (class 4 or class 5) genetic diagnosis in 28% of our patients (Figs. 1c and 2, see Table 1 and Additional file 2: Table S2). In total, 84 (33%) of all 254 patients were referred from Saudi Arabia; the diagnostic yield (57%, 48/84 patients) in this sub-cohort was significantly (*P* value 2.4e−11, two-sided Fisher’s exact test) higher than that in patients of European descent (14%, 24/170). In four of these patients, two independent pathogenic variants in different genes were identified which both contributed to the patient phenotypes (see Additional file 2: Table S2. pt 83.1, 147.1, 153.1, and 222.1).

For an additional 12 patients (5%), exome analysis only identified novel variants in known genes (class 3) with overlapping disease phenotypes (see Additional file 3: Table S3).

### Homozygosity calling

**Fig. 3:**
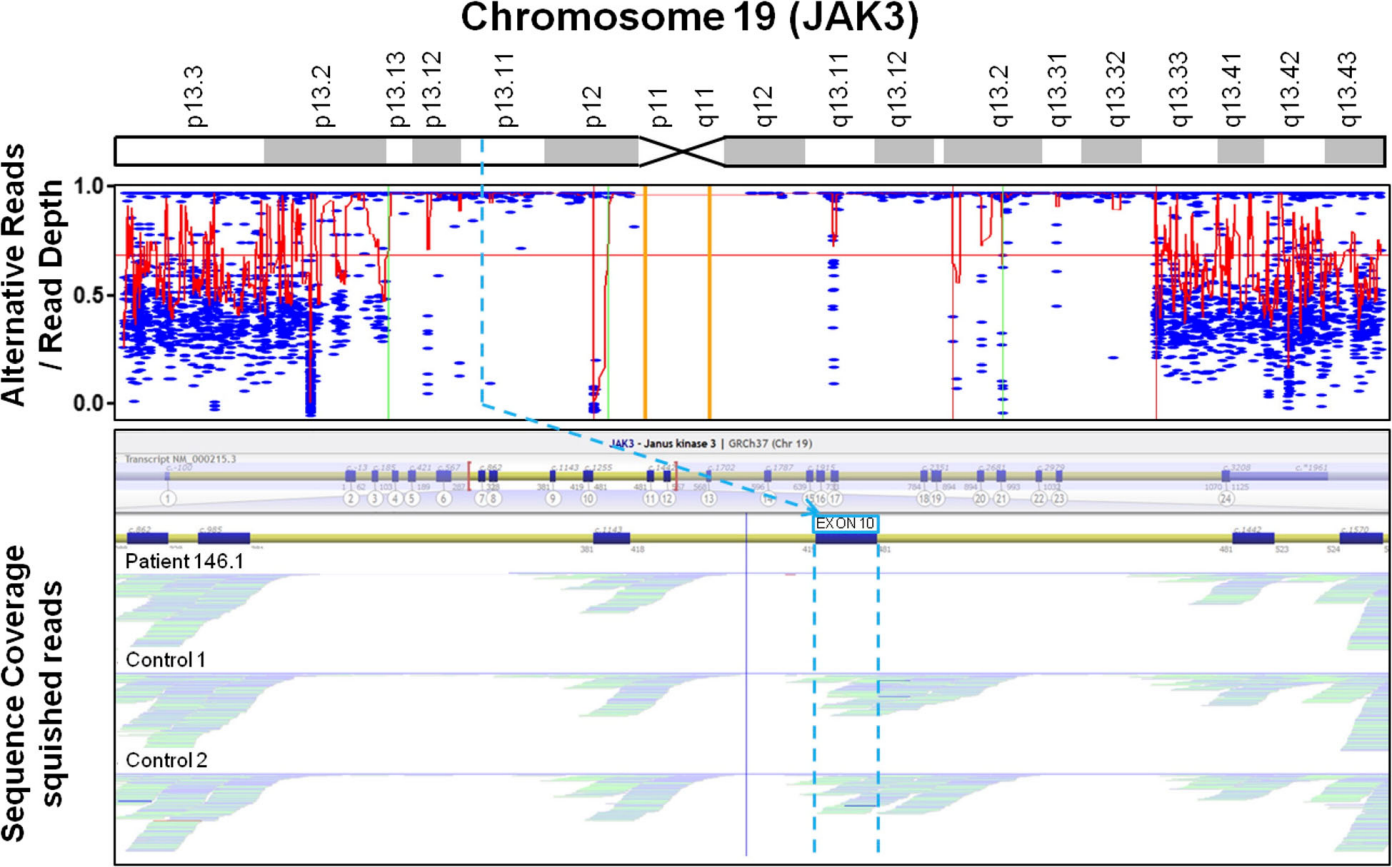
For one Saudi Arabian SCID patient (146.1), exome-based homozygosity mapping identified a large homozygous region on chromosome 19. Further analysis of JAK3 revealed a homozygous deletion of exon 10

We identified in total 1399 large (≥ 5 Mb) homozygous regions in 165 of 254 patient exomes. One thousand sixty-seven of these regions were identified in 81 patients from Saudi Arabia, 318 regions in 68 Dutch patients, and 14 regions in 6 cases from Finland. Thirty-three (82%) of all homozygous pathogenic variants in autosomal recessive genes were present in these homozygous regions. In one Saudi Arabian patient suffering from severe combined immunodeficiency (SCID) (T−, B+, NK−; see Table 1 and Additional file 2: Table S2, 146.1), homozygosity mapping revealed three large homozygous regions on chromosome 19 spanning in total 32.8 Mb (see Additional file 6: Table S6 and Additional file 7: Additional material and references). One of these regions overlapped with the genetic location of *JAK3*, pathogenic variants which are a known cause of SCID [1]. In-depth analysis of *JAK3* resulted in identification of a homozygous deletion of exon 10 (Fig. 3).

### Unclear pathogenic effect of known TRAF3 variants

In five phenotypically heterogeneous patients, exome sequencing identified potentially causative TRAF3 variants; four of our patients carried the heterozygous p.R118W variant which was reported earlier in a patient with herpes simplex encephalitis [27], and one patient carried a p.V240I variant in heterozygous state (see Additional file 3: Table S3. pt 42.1, 76.1, 95.1, 132.1, 209.1).

### Altered therapy options after genetic diagnosis

For 30 (30/72 = 42%) patients for whom exome sequencing molecularly confirmed the diagnosis of SCID, immunodeficiency, centromere instability, and facial anomalies (ICF) syndrome, chronic granulomatous disease, or chronic mucocutaneous candidiasis, bone marrow transplantation is a published treatment option. For additional 25 patients (34%) (Table 1), the genetic diagnosis defined targeted therapeutic options based on available literature [8, 28–52].

## Discussion

The clinical and genetic heterogeneity of PIDs makes exome sequencing a valuable first-tier diagnostic tool for identification of genetic defects underlying PIDs. We present routine diagnostic exome sequencing in a phenotypically heterogeneous group of 254 patients from 249 families. Exome sequencing identified pathogenic genetic variants (interpreted as class 4 or 5) in established disease-causing genes in 72 patients (28%). In four of these patients, a dual genetic diagnosis was made based on two independent genetic pathogenic variants similar to a report for two developmental phenotypes [53].

In addition, for 12 patients (5%), we have identified genetic variants that could possibly contribute to disease, as these patients presented with OMIM-associated clinical features. However, there was insufficient genetic and functional evidence to conclude on the pathogenicity of these variants, which therefore remained variants of unknown significance (class 3 variants; see Additional file 3: Table S3).

The diagnostic yield in our study is in line with other studies describing targeted or exome-wide analyses for heterogeneous groups of PID patients [5, 11, 54, 55]. Phenotypic selection for homogeneous patient cohorts with immunological defects result in increased percentages of diagnoses [56–58]. In addition, the PID-associated genes selected for the gene panels, and the stringency of variant prioritization, result in (minor) differences amongst these studies.

**Fig. 4:**
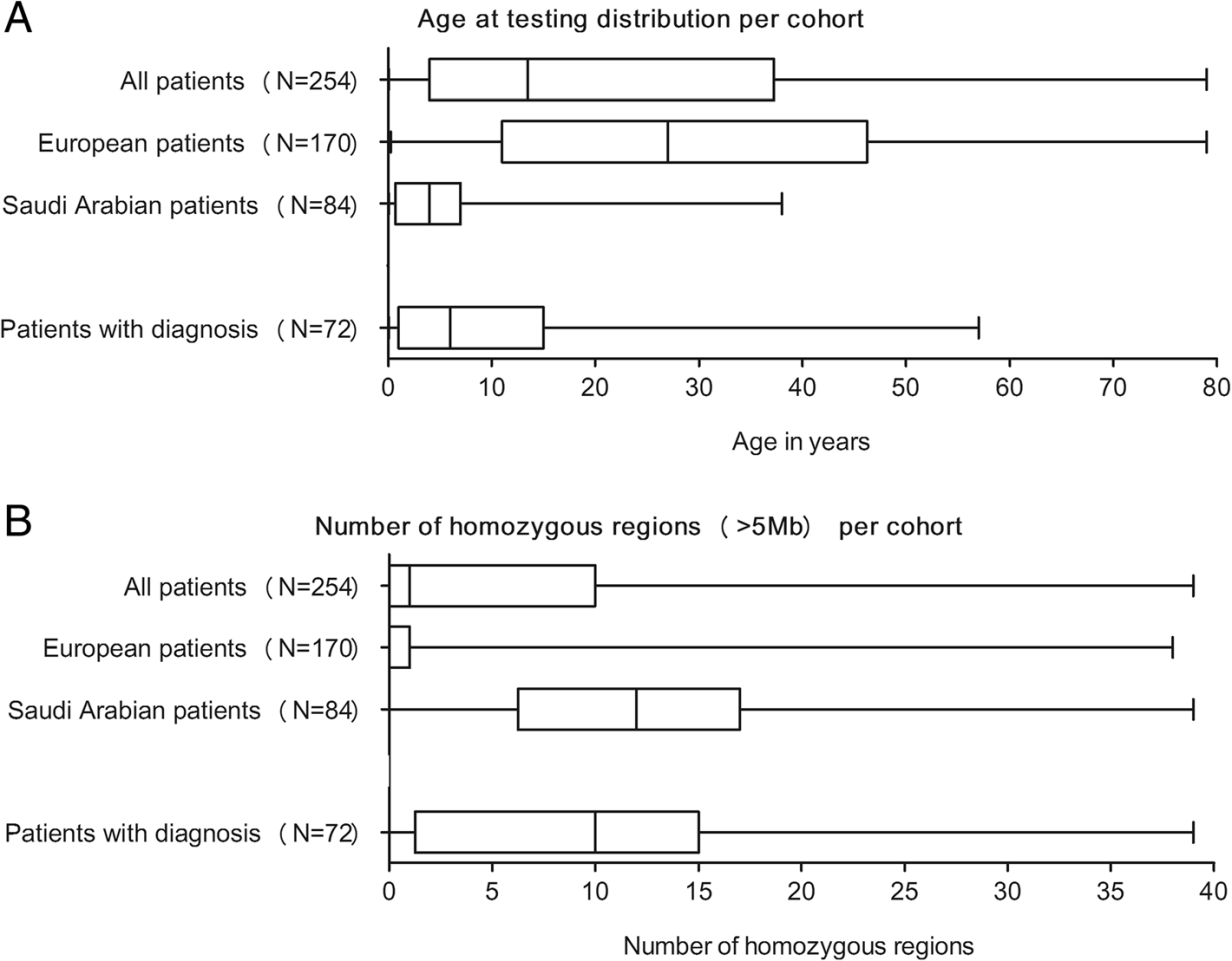
Differences in percentage diagnostic yield based on age and homozygous regions. **a** The age distribution of the entire cohort, the European cohort, the Saudi Arabian cohort, and the cases with a genetic diagnosis. **b** The number of large (> 5 Mb) homozygous regions per cohort. The increased number of homozygous regions in the Saudi Arabian cohort influenced diagnostic yield of the overall cohort

We observed a higher percentage of genetic diagnosis for patients referred from Saudi Arabia (57%) compared to patients from Europe (14%) (Fig. 1c). This significant (*P* value 2.4e−11, two-sided Fisher’s exact test) difference likely arises from two major reasons. Firstly, the Saudi Arabian patients are referred at a very young age (average age of 5.5 years compared to 29.3 years for the European cohort, Fig. 4a), which creates a selection bias towards more severely affected patients. It generally remains challenging to provide molecular diagnoses for older patients from heterogeneous backgrounds. Variants causing late-onset disorders are likely present at higher frequencies in population databases like GnomAD. In addition, the older patients have a higher risk to be exposed to environmental factors (specific pathogens) during their lifetime, highlighting an extra challenge for PID diagnostics.

Secondly, increased consanguinity levels in the Saudi Arabian population create a bias towards homozygously inherited defects. Since the vast majority of known PID genes (69%) cause disease in an AR fashion, higher rates of molecular diagnoses can be expected in patients with more homozygous regions. We detected homozygous pathogenic variants in 44/254 patients, of which 36 were referred from Saudi Arabia. The Saudi Arabian patients have a significantly (*P* value < 0.0001, Welch’s *t*-test) higher number of genomic homozygous regions compared to the European patients (Fig. 4b, see Additional file 6: Table S6).

Not all homozygous pathogenic variants described in this study are SNVs or indels commonly identified by exome sequencing. Homozygosity mapping on exome data can also reveal regions in which homozygous copy number variants (CNVs) may occur. As an example, we focused on a homozygous region on chromosome 19 and could identify a disease-causing homozygous single exon deletion (patient 146.1, *JAK3* exon 10, Fig. 3) in one SCID patient. The contribution of CNVs such as single exon deletions to disease is underestimated in many genetic analyses for PIDs [5], and more systematic assessments from WES data allow up to 6% disease-causing CNVs in heterogeneous disorders [25].

Next to the AR-inherited variants, exome sequencing provided heterozygously rare and private variants that affect known PID (-associated) genes. In case a novel genetic variant did not pass the conservative guideline thresholds [21, 23], the variant was not considered pathogenic in this patient. We acknowledge that these stringent criteria limited the diagnostic outcome of exome sequencing in our cohort at this stage, but we feel this is important in order to prevent misdiagnoses. Systematic trio analysis and functional characterization of each novel missense variant are warranted to gain further insight in the disease mechanism on the individual level [6].

In this study, most pathogenic (class 5) variants were discovered in AR PID genes. There is a bias towards AR disease because all known AR PID genes are caused by genetic loss-of-function (LoF) mutations, and most LoF mutations are considered pathogenic in the ACMG classification [21]. In contrast, the majority of AD-inherited PIDs are the result of gain-of-function (GoF) or dominant-negative mechanisms and are therefore more likely the result of missense variants, or truncating variants affecting the last exon or the last 50 nucleotides of the penultimate exon of the gene [21, 59]. These variants are only considered pathogenic (class 5) in case the exact same genetic variant was described earlier. Generally, these types of variants are less likely pathogenic since the altered RNA is not predicted to undergo nonsense-mediated decay and the altered protein is expressed [60].

Exome sequencing analysis identified more pathogenic variants in specific subgroups of patients compared to others. Similar to earlier reports, severely affected patients were more likely to receive a genetic diagnosis [5]. First, evaluation of clinical characteristics revealed that the patients with a higher burden of infections caused by multiple pathogens and/or autoimmune manifestations were significantly (*P* value 0.0002, two-sided Fisher’s exact test) more likely to receive a genetic diagnosis (40%; 48/121), compared to patients with infections restricted to a single pathogen or autoimmune manifestation (14%; 14/98) (Fig. 1a). Moreover, patients with defects in important immune cell populations are expected to have more clinical manifestations. In line with this, a significantly (*P* value 0.0014, two-sided Fisher’s exact test) higher percentage of patients with aberrant blood cell populations received a genetic diagnosis (37%; 49/133), compared to patients with normal blood cell populations (19%; 23/123) (Fig. 1b).

### Unclear pathogenic effect of known TRAF3 variants

The exact same pathogenic variants as previously reported in literature were identified in 40 patients from our cohort. In 36 (90%) of these patients, the presented immunophenotypic characteristics were similar to earlier described cases. However, exome sequencing revealed the same TRAF3 variant p.(R118W) as described in one patient with HSV encephalitis in four patients (see Additional file 3: Table S3. 44.1, 76.1, 99.1, and 217.1) within our cohort [27]. Without careful phenotypic assessment and genetic evaluation, this may result in a false diagnosis. Only one of the patients carrying a (paternally inherited) TRAF3 variant (76.1) suffered from HSV infections, which could also be caused by a (maternally inherited) frameshift variant in GATA2 p.(R86fs/wt) [61]. In addition, this variant is relatively common in the population [20] (population frequency of 0.3%) and appeared slightly more frequent in this study (1.5%). Due to the high population frequency and disease heterogeneity, we speculate that the TRAF3 variant p.(R118W) might result in a minor broad immunomodulatory defect, and additional genetic and environmental factors further determine the clinical presentation. We therefore concluded that this specific variant was not solely the cause of disease in these patients but should be rather considered a risk/susceptibility factor. This may be important for future diagnostic interpretation of this variant.

### Diagnosis by exome-wide analysis

One hundred fifty-six diagnosis-negative patients provided additional informed consent for exome-wide analysis of their data. For 10 of these patients, we identified disease-causing variants in genes that were described after the latest gene panel update, or genes known to affect specific sub-pathways that have been previously published as a genetic cause of similar phenotypes (Table 1). This is exemplified by one case (70.1) suffering from recurrent respiratory tract infections for which exome sequencing identified a homozygous pathogenic variant in the first amino acid of the protein *RSPH9* p.(M1T/M1T) [50]. The genotype-first approach led to identification of the pathogenic variant leading to ciliary dyskinesia in this patient, which retrospectively fits the clinical diagnosis. Re-analysis of exome data for novel disease genes, as well as further functional, co-segregation and overlap analysis will ultimately lead to additional genetic diagnoses for a subset of these patients.

### Genetic diagnosis-based treatment options

In total, 24 patients were molecularly diagnosed with severe immunological phenotypes like severe combined immunodeficiency (SCID) or immunodeficiency, centromere instability, and facial anomalies (ICF) syndrome, for which bone marrow transplantation is the main remedy [8]. This treatment option may have been considered already based on the clinical presentation alone for some cases; however, referring clinicians valued the molecular diagnosis of SCID confirming this treatment options.

In addition, six patients were molecularly diagnosed with chronic mucocutaneous candidiasis (CMC) or chronic granulomatous disease (CGD), which can be treated with ruxolitinib (CMC) or IFN-γ (CGD) [46, 62]. However, HSCT has also been published as a therapeutic option for these diseases [35, 47].

For an additional 25 patients, the genetic diagnosis provided novel options for targeted therapeutics based on recent literature (Table 1) [8, 28–52]. The long-term effect of these altered therapeutic strategies is still unknown.

## Conclusions

In conclusion, exome sequencing proves to be a valuable first-tier test for routine diagnostics in PIDs providing a genetic diagnosis in 28% of patients. In addition, exome sequencing harbors advantages over gene panels as a truly generic test for all genetic diseases, including in silico extension of existing gene lists and re-analysis of the existing data whenever new knowledge is available.

Importantly, we observed that identifying the molecular diagnosis in PID patients confirmed HSCT in 42% of cases as a possible treatment option and identified therapeutic target options for additional 34% of cases. This high amount of possibly “actionable mutations” is uncommon for genetic disorders due to germline mutations, but highlights the possibilities for PIDs in truly personalized medicine. Future studies combining systematic trio analysis of exome, genome, and/or transcriptome data will provide patients with additional diagnoses and insights in targeted therapeutics.

## Additional files

Additional file 1: **Table S1.** Overview of all clinical characteristics of the patients included in our diagnostic PID cohort, including all immunophenotype characteristics. (XLSX 63 kb)

Additional file 2: **Table S2.** Shows all causative mutations identified in 72 patients from 68 families suffering from primary immunodeficiencies. (XLSX 17 kb)

Additional file 3: **Table S3.** Variants of unknown significance (class 3) and variants in TRAF3 identified in 17 patients suffering from primary immunodeficiencies. (XLSX 11 kb)

Additional file 4: **Table S4.** (A) The number of patients with isolated or combined infections, and (B) the number of patients with isolated or combined immunophenotypes, and the percentage for which we have reported a genetic diagnosis. (XLSX 11 kb)

Additional file 5: **Table S5.** Quality information of the WES technology, with the mean target coverage, and the % of bases with > 20× coverage. (XLSX 22 kb)

Additional file 6: **Table S6.** Information on all large > 5-Mb homozygous regions per patient, detected in the exome. Of each region, the genomic location, size, % homozygous variants, and the detected mutation are provided. (XLSX 158 kb)

Additional file 7: Additional material and references. (DOCX 31 kb)

## Data Availability

The datasets supporting the conclusions of this article are included within the article and its additional files. All raw data was retrieved in the realm of patients’ diagnostic procedure; this does not allow sharing of the data publically, because the patient families were not consented for sharing their raw data, which can potentially identify the individuals.
