# Supplementary material for: Exome sequencing in routine diagnostics: a generic test for 254 patients with primary immunodeficiencies
Source: Genome Med. 2019 Jun 17;11:38. doi: 10.1186/s13073-019-0649-3 (PMC6572765; doi:10.1186/s13073-019-0649-3)
Supplement: Supplementary file 7 — Additional material and references. (DOCX 31 kb) [file 13073_2019_649_MOESM7_ESM.docx]

**Additional material descriptions:**

**Additional file 1. Overview of all clinical characteristics of the patients included in our diagnostic PID cohort, including all immunophenotype characteristics.**

**Bacterial acronyms:** AB = Acinetobacter Baumannii; BCG = Bacillus Calmette-Guérin; BAF = Bacteroide fragalis; BHSC = Beta-hemolytic Streptococcus group C; BOP = Bortedella Pertussis; BP = Burkholderia Pseudomallei; CDF = Clostridium Difficile; COB = Coxiella burnetii (Q-fever); EC = Escherchia Coli; ENC = Enterobacter Cloacae; ENG = Enterobacter Gergoviae; EFM = Enterococcus Faecium; EFS = Enterococcus Faecalis; GBS = Group B Streptococcus; HI = Heamophilus Influenzae; HPI = Heamophilus Parainfluenzae; KOX = Klebsiella Oxytoca; KP = Klebsiella Pneumoniae; MC = Moraxella catarrhalis; MRSA = Methicilin-Resistant Staphylococcus Aureus; MY = Mycobacteria; NM = Neisseria Meningitidis; PRB = Proprionibacterium ; PRT = Proteus; PSA = Pseudomonas Aeruginosa; SA = Staphylococcus Aureus; SE = Staphylococcus Epidermidis; SH = Staphylococcus Hominis; SHC = Staphylococcus Heamolyticus; SM = Serratia Marcescens; SAL = Salmonella; SO = Streptococcus Oralis; SP = Streptococcus Pneumoniae; SV = Streptococcus Viridans; TW = Tropheryma Whipplei; X = Not specified

**Viral acronyms:** CMV = CytoMegaloVirus; EBV = Epstein Barr Virus; HAdV = Human AdenoVirus; HBoV = Human BocaVirus; HCoV = Human CoronaVirus; HPV = Human Pappiloma Virus; HPIV = Human ParaInfluenzaVirus; HRV = Human RhinoVirus; HSV = Herpes Simplex Virus; IFV = Influenza Virus; RSV = Respiratory Syncytial Virus; RV = Rotavirus; VZV = Varicella Zoster Virus; A = Type A; B = Type B; 1 = Type 1; 2 = Type 2; 3 = Type 3; 4 = Type 4; X = Not specified

**Fungal or parasitic acronyms:** ^A^ = Candida Albicans; ^G^ = Glabrata; CA^T^ = Tropicalis; DF = Dientamoeba Fragalis; MF = Malassezia Furfur; PJ = Pneumocystis Jirovecii; X = Not specified

**Autoimmune or autoinflammatory acronyms:** AA = Alopecia Areata ; AIM = Autoimmune; AIF = Autoinflammatory; ANCASV = Anti-Neutrophil Cytoplasmic Antibody systemic vasculitis ; ALPS = Autoimmune Lymphoproliferative Syndrome; APS = Antiphospholipid syndrome; ARTH = Arthritis; ARTHA = Aseptic Arthritis ; ARTHPJ = Polyarthritis Juvenalis; CD = Celiac Disease ; DM1 = Diabetes Mellitus type 1; EnP = Enteropathy; GLILD = Granulomatous-Lymphocytic Interstitial Lung Disease ; Hypo = Hypothyroidism; IBD = Inflammatory Bowel Disease; IleitisT = Ileitis Terminalis; LAD = Lymphadenopathy; NP = Nephropathy; SLE = Systemic Lupus Erythematosus; SSc = Systemic Sclerosis; X = Not specified

**Infection site acronyms:** CNS = Central Nervous System; ENT = Ear Nose Throat

**Additional features acronyms:** ADHD = Attention Deficit Hyperactivity Disorder; ASD = Autism Spectrum Disorder; BMF = Bone Marrow Failure; CHD = Coronary Heart Disease; DD = Developmental Delay; EPI = Exocrine Pancreatic Insufficiency; FD = Feeding Difficulties; FTT = Failure To Thrive; GERD = Gastroesophagal Reflux Disease; †= patient deceased

**Expected disease acronyms:** ALPS = Autoimmune LymphoProliferative Syndrome; APECED = Autoimmune PolyEndocrinopathy-Candidiasis-Ectodermal-Dystrophy; CVID = Common Variable Immunodeficiency; CMC = Chronic Mucocutaneous Candidiasis; GLILD= Granulomatous Lymphocytic Interstitial Lung Disease; HGG = Hypogammaglobulinemia; HLH = Hemophagocytic Lymphohistiocytosis; HSV = Herpes Simplex Virus; IPEX = Immunodysregulation Polyendocrinopathy Enteropathy X-linked syndrome; MSMD = Mendelian Susceptibility to Mycobacterial Diseases; PAPA = Pyogenic Arthritis Pyoderma gangrenosum and Acne; PFAPA = Periodic Fever Aphthous Stomatitis Pharyngitis Adenitis Syndrome; SCID = Severe Combined Immunodeficiency; SLE= Systemic Lupus Erythematosus; TORCH = toxoplasmosis, other infections, rubella, cytomegalovirus infection, and herpes simplex

**Indications of Cytopenias:** A = Aplastic anemia; AIH = AutoImmune Hemolytic Anemia; CN = Cyclic neutropenia; EO = Eosinopenia; H = Hemolytic anemia; IP = Immune thrombocytopenic purpura; N = Neutropenia; NC = Normocytic anemia; TR = Transient; X = Yes, type not specified

**Additional file 2. Shows all causative mutations identified in 72 patients from 68 families suffering from primary immunodeficiencies.** These 72 are part of a diagnostically tested cohort of 254 PID patients from 249 families. The “variant class” column also indicates variants that were identified by exome-wide analysis. ‡the homozygous mutation was identified in one of the large homozygous regions. Abbreviations used: AR = Autosomal Recessive; AD = Autosomal Dominant; XL = X-linked; hom = homozygous; CH = Compound heterozygous; APECED = Autoimmune PolyEndocrinopathy-Candidiasis-Ectodermal-Dystrophy; CVID = Common Variable Immunodeficiency; HLH = Hemophagocytic Lymphohistiocytosis; PAPA = Pyogenic Arthritis Pyoderma gangrenosum and Acne; SCID = Severe Combined Immunodeficiency; TORCH = toxoplasmosis, other infections, rubella, cytomegalovirus infection, and herpes simplex

**Additional file 3. Shows variants of unknown significance (class 3) and variants in TRAF3 identified in 17 patients suffering from primary immunodeficiencies.** These 17 are part of a diagnostically tested cohort of 254 PID patients from 249 families. The “variant class” column also indicates variants that were identified by exome-wide analysis.*Only 1 (76.1) of 4 patients carrying a variant in TRAF3 suffered from infections with herpes simplex virus, which might also result form a mutation in GATA2 p.(R86fs/wt). ‡the homozygous mutation was identified in one of the large homozygous regions. Abbreviations used: AR = Autosomal Recessive; AD = Autosomal Dominant; XL = X-linked; hom = homozygous; CH = Compound heterozygous

**Additional file 4.** Provides **(A)** the number of patients with isolated or combined infections, and **(B)** the number of patients with isolated or combined immunophenotypes, and the percentage for which we have reported a genetic diagnosis

**Additional file 5.**

Provides quality information of the WES technology, with the mean target coverage, and the % of bases with >20X coverage.

**Additional file 6.**

Provides information on all large >5mb homozygous regions per patient, detected in the exome. Of each region, the genomic location, size, % homozygous variants, and the detected mutation is provided.

**References for additional files 2 and 3.**

S1. Nagamine K, Peterson P, Scott HS, et al. Positional cloning of the APECED gene. Nat. Genet. 1997; 17:393–398.

S2. Demidowich AP, Freeman AF, Kuhns DB, et al. Genotype, Phenotype, and Clinical Course in Five Patients With PAPA Syndrome (Pyogenic Sterile Arthritis, Pyoderma Gangrenosum, and Acne). Arthritis Rheumatol. 2012; 64:2022–2027.

S3. Dell’Angelica EC, Shotelersuk V, Aguilar RC, Gahl WA, Bonifacino JS. Altered trafficking of lysosomal proteins in Hermansky-Pudlak syndrome due to mutations in the ??3A subunit of the AP-3 adaptor. Mol. Cell 1999; 3:11–21.

S4. Clark RH, Stinchcombe JC, Day A, et al. Adaptor protein 3-dependent microtubule-mediated movement of lytic granules to the immunological synapse. Nat. Immunol. 2003; 4:1111–1120.

S5. Hernandez PA, Gorlin RJ, Lukens JN, et al. Mutations in the chemokine receptor gene CXCR4 are associated with WHIM syndrome, a combined immunodeficiency disease. Nat Genet 2003; 34:70–74.

S6. Veerdonk FL van de, Plantinga TS, Alexander Hoischen, et al. STAT1 mutations in autosomal dominant chronic mucocutaneous candidiasis. N. Engl. J. Med. 2011; 365:54–61.

S7. Liu L, Okada S, Kong X-F, et al. Gain-of-function human STAT1 mutations impair IL-17 immunity and underlie chronic mucocutaneous candidiasis. J. Exp. Med. 2011; 208:1635–1648.

S8. Ly H, Schertzer M, Jastaniah W, et al. Identification and functional characterization of 2 variant alleles of the telomerase RNA template gene (TERC) in a patient with dyskeratosis congenita. Blood 2005; 106:1246–1252.

S9. Schubert D, Bode C, Kenefeck R, et al. Autosomal dominant immune dysregulation syndrome in humans with CTLA4 mutations. Nat. Med. 2014; 20:1410–6.

S10. Ng SB, Bigham AW, Buckingham KJ, et al. Exome sequencing identifies MLL2 mutations as a cause of Kabuki syndrome. Nat. Genet. 2010; 42:790–793.

S11. Makrythanasis P, van Bon BW, Steehouwer M, et al. MLL2 mutation detection in 86 patients with Kabuki syndrome: A genotype-phenotype study. Clin. Genet. 2013; 84:539–545.

S12. Castleman VH, Romio L, Chodhari R, et al. Mutations in radial spoke head protein genes RSPH9 and RSPH4A cause primary ciliary dyskinesia with central-microtubular-pair abnormalities. Am. J. Hum. Genet. 2008; 84:197–209.

S13. Hsu AP, Sampaio EP, Khan J, et al. Mutations in GATA2 are associated with the autosomal dominant and sporadic monocytopenia and mycobacterial infection (MonoMAC) syndrome. Blood 2011; 118:2653–2655.

S14. Lopez-Herrera G, Tampella G, Pan-Hammarström Q, et al. Deleterious mutations in LRBA are associated with a syndrome of immune deficiency and autoimmunity. Am. J. Hum. Genet. 2012; 90:986–1001.

S15. Wiszniewski W, Fondaneche MC, Louise-Plence P, et al. Novel mutations in the RFXANK gene: RFX complex containing in-vitro-generated RFXANK mutant binds the promoter without transactivating MHC II. Immunogenetics 2003; 54:747–755.

S16. Højlund K, Hansen T, Lajer M, et al. A novel syndrome of autosomal-dominant hyperinsulinemic hypoglycemia linked to a mutation in the human insulin receptor gene. Diabetes 2004; 53:1592–1598.

S17. Rice GI, del Toro Duany Y, Jenkinson EM, et al. Gain-of-function mutations in IFIH1 cause a spectrum of human disease phenotypes associated with upregulated type I interferon signaling. Nat. Genet. 2014; 46:503–9.

S18. Ravenscroft JC, Suri M, Rice GI, Szynkiewicz M, Crow YJ. Autosomal dominant inheritance of a heterozygous mutation in SAMHD1 causing familial chilblain lupus. Am. J. Med. Genet. Part A 2011; 155:235–237.

S19. Apostolou S, Whitmore S, Crawford J, et al. Positional cloning of the Fanconi anaemia group A gene. Nat Genet. 1996; 14:353–6.

S20. Lucas CL, Kuehn HS, Zhao F, et al. Dominant-activating germline mutations in the gene encoding the PI(3)K catalytic subunit p110 δ result in T cell senescence and human immunodeficiency. Nat. Immunol. 2014; 15:88.

S21. Deau MC, Heurtier L, Frange P, et al. A human immunodeficiency caused by mutations in the PIK3R1 gene. J. Clin. Invest. 2014; 124:3923–3928.

S22. Fliegauf M, L. Bryant V, Frede N, et al. Haploinsufficiency of the NFκB1 Subunit p50 in Common Variable Immunodeficiency. Am. J. Hum. Genet. 2015; 97:389–403.

S23. Puck JM, Pepper a E, Henthorn PS, et al. Mutation analysis of IL2RG in human X-linked severe combined immunodeficiency. Blood 1997; 89:1968–77.

S24. Schwarz K, Gauss GH, Ludwig L, et al. RAG mutations in human B cell-negative SCID. Science 1996; 274:97–99.

S25. Derry JM, Ochs HD, Francke U. Isolation of a novel gene mutated in Wiskott-Aldrich syndrome. Cell 1994; 78:635–644.

S26. Proust A, Guillet B, Pellier I, et al. Recurrent V75M mutation within the Wiskott-Aldrich syndrome protein: Description of a homozygous female patient. Eur. J. Haematol. 2005; 75:54–59.

S27. Zhong F, Savage S a, Shkreli M, et al. Disruption of telomerase trafficking by TCAB1 mutation causes dyskeratosis congenita service Disruption of telomerase trafficking by TCAB1 mutation causes dyskeratosis congenita. Genes Dev. 2011; 25:11–16.

S28. Moshous D, Callebaut I, De Chasseval R, et al. Artemis, a novel DNA double-strand break repair/V(D)J recombination protein, is mutated in human severe combined immune deficiency. Cell 2001; 105:177–186.

S29. Arpaia E, Shahar M, Dadi H, Cohen A, Rolfman CM. Defective T cell receptor signaling and CD8+ thymic selection in humans lacking Zap-70 kinase. Cell 1994; 76:947–958.

S30. Zhou Q, Yang D, Ombrello AK, et al. Early-onset stroke and vasculopathy associated with mutations in ADA2. N. Engl. J. Med. 2014; 370:911–20.

S31. Newman PJ, Seligsohn U, Lyman S, Coller BS. The molecular genetic basis of Glanzmann thrombasthenia in the Iraqi-Jewish and Arab populations in Israel. Proc. Natl. Acad. Sci. U. S. A. 1991; 88:3160–3164.

S32. Buitrago L, Rendon A, Liang Y, et al. αIIbβ3 variants defined by next-generation sequencing: predicting variants likely to cause Glanzmann thrombasthenia. Proc. Natl. Acad. Sci. U. S. A. 2015; 112:E1898-907.

S33. Xu GL, Bestor TH, Bourc’his D, et al. Chromosome instability and immunodeficiency syndrome caused by mutations in a DNA methyltransferase gene. Nature 1999; 402:187–191.

S34. Kaya N, Al-Muhsen S, Al-Saud B, et al. ICF syndrome in Saudi Arabia: Immunological, cytogenetic and molecular analysis. J. Clin. Immunol. 2011; 31:245–252.

S35. De Greef JC, Wang J, Balog J, et al. Mutations in ZBTB24 are associated with immunodeficiency, centromeric instability, and facial anomalies syndrome type 2. Am. J. Hum. Genet. 2011; 88:796–804.

S36. Conrad M, Noor D, Sullivan K, Devoto M, Kelsen J. Identification of a Homozygous Mutation in the ZBTB24 Gene in a Patient with Very Early Onset Inflammatory Bowel Disease. Pedriatic poster Present. 2016; :2016.

S37. Dinauer MC, Pierce E a, Bruns G a P, Cumutte JT, Orkin SH. Human Neutrophil Cytochrome b Light Chain (p22-phox). J. Clin. Cinvest. 1990; 86:1729–1737.

S38. Pannicke U, Hönig M, Hess I, et al. Reticular dysgenesis (aleukocytosis) is caused by mutations in the gene encoding mitochondrial adenylate kinase 2. Nat. Genet. 2009; 41:101–105.

S39. Al-Mousa H, Abouelhoda M, Monies DM, et al. Unbiased targeted next-generation sequencing molecular approach for primary immunodeficiency diseases. J. Allergy Clin. Immunol. 2016; 137:1780–1787.

S40. Kerem B, Rommens JM, Buchanan JA, et al. Identification of the Cystic Fibrosis Gene : Identification Genetic Analysis. Science (80-. ). 1989; 245.

S41. Vidaud M, Fanen P, Martin J, Ghanem N, Nicolas S, Goossens M. Three point mutations in the CFTR gene in French cystic fibrosis patients: identification by denaturing gradient gel electrophoresis. Hum. Genet. 1990; 85:446–9.

S42. Macchi P, Villa a, Giliani S, et al. Mutations of Jak-3 gene in patients with autosomal severe combined immune deficiency (SCID). Nature. 1995; 377:65–68.

S43. Scarselli A, Di Cesare S, Di Matteo G, et al. Combined immunodeficiency due to JAK3 mutation in a child presenting with skin granuloma. J. Allergy Clin. Immunol. 2016; 137:948–951.

S44. Chun HJ, Zheng L, Ahmad M, et al. Pleiotropic defects in lymphocyte activation caused by caspase-8 mutations lead to human immunodeficiency. Nature 2002; 419:395–399.

S45. Niemeyer CM, Kang MW, Shin DH, et al. Germline CBL mutations cause developmental abnormalities and predispose to juvenile myelomonocytic leukemia. Nat. Genet. 2010; 42:794–800.

S46. Kojima T, Horiuchi T, Nishizaka H, et al. Genetic basis of human complement C8 alpha-gamma deficiency. J. Immunol. 1998; 161:3762–3766.

S47. Varon R, Vissinga C, Platzer M, et al. Nibrin, a novel DNA double-strand break repair protein, is mutated in Nijmegen breakage syndrome. Cell 1998; 93:467–476.

S48. Gazda HT, Sheen MR, Vlachos A, et al. Ribosomal Protein L5 and L11 Mutations Are Associated with Cleft Palate and Abnormal Thumbs in Diamond-Blackfan Anemia Patients. Am. J. Hum. Genet. 2008; 83:769–780.

S49. Vilboux T, Lev A, Malicdan MC V., et al. A Congenital Neutrophil Defect Syndrome Associated with Mutations in VPS45. N. Engl. J. Med. 2013; 369:54–65.

S50. Stepp SE, Dufourcq-Lagelouse R, Le Deist F, et al. Perforin gene defects in familial hemophagocytic lymphohistiocytosis. Science (80-. ). 1999; 286:1957–1959.

S51. Castigli E, Wilson SA, Garybyan L, et al. TACI mutation in common variable immunodeficiency and IgA deficiency. Nat. Genet. 2006; 6:357–362.

S52. Lekstrom-Himes JA, Dorman SE, Kopar P, Holland SM, Gallin JI. Neutrophil-specific granule deficiency results from a novel mutation with loss of function of the transcription factor CCAAT/enhancer binding protein e. J. Exp. Med. 1999; 189:1847–1852.

S53. Köker MY, Camcıoğlu Y, van Leeuwen K, et al. Clinical, functional, and genetic characterization of chronic granulomatous disease in 89 Turkish patients. J. Allergy Clin. Immunol. 2013; 132:1156–1163.e5.

S54. Li F-Y, Chaigne-Delalande B, Kanellopoulou C, et al. Second messenger role for Mg2+ revealed by human T-cell immunodeficiency. Nature 2011; 475:471–476.

S55. Zhong FL, Mamaï O, Sborgi L, et al. Germline NLRP1 Mutations Cause Skin Inflammatory and Cancer Susceptibility Syndromes via Inflammasome Activation. Cell 2016; 167:187–202.e17.

S56. Villard J, Reith W, Barras E, et al. Analysis of mutations and chromosomal localisation of the gene encoding RFX5, a novel transcription factor affected in major histocompatibility complex class II deficiency. Hum. Mutat. 1997; 10:430–435.

S57. Atanasovska B, Bozhinovski G, Plaseska-Karanfilska D, Chakalova L. Efficient Detection of Mediterranean β-Thalassemia Mutations by Multiplex Single-Nucleotide Primer Extension. PLoS One 2012; 7.

S58. Crow YJ, Leitch A, Hayward BE, et al. Mutations in genes encoding ribonuclease H2 subunits cause Aicardi-Goutières syndrome and mimic congenital viral brain infection. Nat. Genet. 2006; 38:910–6.

S59. Nishizaka H, Horiuchi T, Zhu ZB, Fukumori Y, Volanakis JE. Genetic bases of human complement C7 deficiency. J. Immunol. 1996; 157:4239–4243.

S60. Verhoeven NM, Huck JH, Roos B, et al. Transaldolase deficiency: liver cirrhosis associated with a new inborn error in the pentose phosphate pathway. Am. J. Hum. Genet. 2001; 68:1086–1092.

S61. Eyaid W, Al Harbi T, Anazi S, et al. Transaldolase deficiency: Report of 12 new cases and further delineation of the phenotype. J. Inherit. Metab. Dis. 2013; 36:997–1004.

S62. Hauck F, Randriamampita C, Martin E, et al. Primary T-cell immunodeficiency with immunodysregulation caused by autosomal recessive LCK deficiency. J. Allergy Clin. Immunol. 2012; 130.

S63. Snow AL, Xiao W, Stinson JR, et al. Congenital B cell lymphocytosis explained by novel germline CARD11 mutations. J Exp Med 2012; 209:2247–2261.

S64. Brohl AS, Stinson J, Su HC, et al. Germline CARD11 mutation in a patient with severe congenital B cell lymphocytosis. J. Clin. Immunol. 2016; 35:32–46.

S65. Banka S, Blom HJ, Walter J, et al. Identification and characterization of an inborn error of metabolism caused by dihydrofolate reductase deficiency. Am. J. Hum. Genet. 2011; 88:216–225.

S66. Nunoi H, Iwata M, Tatsuzawa S, et al. AG dinucleotide insertion in a patient with chronic granulomatous disease lacking cytosolic 67-kD protein. Blood 1995; 86:329–33.

S67. Aksentijevich I, Torosyan Y, Samuels J, et al. Mutation and haplotype studies of familial Mediterranean fever reveal new ancestral relationships and evidence for a high carrier frequency with reduced penetrance in the Ashkenazi Jewish population. Am. J. Hum. Genet. 1999; 64:949–62.

S68. Romberg N, Al Moussawi K, Nelson-Williams C, et al. Mutation of NLRC4 causes a syndrome of enterocolitis and autoinflammation. Nat. Genet. 2014; 46:1135–9.

S69. Volker-Touw N, De Koning H, Van Kempen T, et al. A novel mutation in NLRC4 in a large pedigree with an anakinra responsive autoinflammatory disease. Pediatr. Rheumatol. 2015; 13:P30.

S70. Vulliamy TJ, D’Urso M, Battistuzzi G, et al. Diverse point mutations in the human glucose-6-phosphate dehydrogenase gene cause enzyme deficiency and mild or severe hemolytic anemia. Proc. Natl. Acad. Sci. USA 1988; 85:5171–5175.

S71. Jarolim P, Rubin HL, Brabec V, Palek J. A Nonsense Mutation 1669Glu→Ter within the Regulatory Domain of Human Erythroid Ankyrin Leads to a Selective Deficiency of the Major Ankyrin Isoform (Band 2.1) and a Phenotype of Autosomal Dominant Hereditary Spherocytosis. J. Clin. Invest. 1995; 95:941–947.

S72. Keller MD, Ganesh J, Heltzer M, et al. Severe combined immunodeficiency resulting from mutations in MTHFD1. Pediatrics 2013; 131:e629-34.

S73. Burda P, Kuster A, Hjalmarson O, et al. Characterization and review of MTHFD1 deficiency: four new patients, cellular delineation and response to folic and folinic acid treatment. J. Inherit. Metab. Dis. 2015; 38:863–872.

S74. Minegishi Y, Saito M, Tsuchiya S, et al. Dominant-negative mutations in the DNA-binding domain of STAT3 cause hyper-IgE syndrome. Nature 2007; 448:1058–1062.

S75. Chandesris M-O, Melki I, Natividad A, et al. Autosomal Dominant STAT3 Deficiency and Hyper-IgE Syndrome Molecular, Cellular, and Clinical Features From a French National Survey. Medicine (Baltimore). 2013; 91.

S76. Boisson B, Laplantine E, Dobbs K, et al. Human HOIP and LUBAC deficiency underlies autoinflammation, immunodeficiency, amylopectinosis, and lymphangiectasia. J. Exp. Med. 2015; 212:939–951.

S77. Bustamante J, Arias A a, Vogt G, et al. Germline CYBB mutations that selectively affect macrophages in kindreds with X-linked predisposition to tuberculous mycobacterial disease. Nat. Immunol. 2011; 12:213–21.

S78. Riley LG, Cooper S, Hickey P, et al. Mutation of the mitochondrial tyrosyl-tRNA synthetase gene, YARS2, causes myopathy, lactic acidosis, and sideroblastic anemia - MLASA syndrome. Am. J. Hum. Genet. 2010; 87:52–59.

S79. Pérez de Diego R, Sancho-Shimizu V, Lorenzo L, et al. Human TRAF3 adaptor molecule deficiency leads to impaired Toll-like receptor 3 response and susceptibility to herpes simplex encephalitis. Immunity 2010; 33:400–411.

S80. Gerin I, Veiga-Da-Cunha M, Achouri Y, Collet JF, Van Schaftingen E. Sequence of a putative glucose 6-phosphate translocase, mutated in glycogen storage disease type Ib. FEBS Lett. 1997; 419:235–238.

S81. Burg M Van Der, Ijspeert H, Verkaik NS, et al. A DNA-PKcs mutation in a radiosensitive T–B– SCID patient inhibits Artemis activation and nonhomologous end-joining. J. Clin. Invest. 2009; 119:91–98.

S82. Dobbs K, Domínguez Conde C, Zhang S-Y, et al. Inherited DOCK2 Deficiency in Patients with Early-Onset Invasive Infections. N. Engl. J. Med. 2015; 372:2409–22.

S83. Fisher GH, Rosenberg FJ, Straus SE, et al. Dominant Interfering Fas Gene-Mutations Impair Apoptosis in a Human Autoimmune Lymphoproliferative Syndrome. Cell 1995; 81:935–946.

S84. Liang J, Yagasaki H, Kamachi Y, et al. Mutations in telomerase catalytic protein in Japanese children with aplastic anemia. Haematologica 2006; 91:656–658.
